# OPN Deficiency Increases the Severity of Osteoarthritis Associated with Aberrant Chondrocyte Senescence and Apoptosis and Upregulates the Expression of Osteoarthritis-Associated Genes

**DOI:** 10.1155/2020/3428587

**Published:** 2020-10-22

**Authors:** Jian Tian, Chao Cheng, Shi-Da Kuang, Chao Su, Xin Zhao, Yi-lin Xiong, Yu-Sheng Li, Shu-Guang Gao

**Affiliations:** ^1^Department of Orthopaedics, Xiangya Hospital, Central South University, 87 Xiangya Road, Changsha 410008, Hunan, China; ^2^Department of Orthopaedics, Yiyang Central Hospital, 118 North KangFu Road, Yiyang, Hunan 413000, China; ^3^National Clinical Research Center of Geriatric Disorders, Xiangya Hospital, Central South University, Changsha, China

## Abstract

**Objectives:**

A recent work has reported that the elevated osteopontin (OPN) levels in the articular cartilage and synovial fluid are correlated with the progressive osteoarthritis (OA) joint damage, and OPN has a protective effect against OA by suppressing the expressions of OA-associated genes. The present study examined whether the OPN deficiency was susceptible to OA through the regulation of chondrocyte senescence and apoptosis and the expressions of OA-associated genes.

**Methods:**

The mRNA levels of COL2A1 and OPN were compared between human OA chondrocytes and normal chondrocytes. The effects of OPN siRNA on the SA-*β*-Gal expressions and the percentage of apoptotic chondrocytes were examined by using SA-*β*-Gal staining and apoptosis assay, and the effects on the expressions of COL2A1 and OA-associated genes (COL10A1, IL-1*β*, TNF-ɑ, MMP-13, and ADAMTS5) were examined by western blot analysis and quantitative real-time RT-PCR. Furthermore, an in vivo OA model was established to examine the effects of OPN siRNA on the senescence and apoptosis of OA chondrocytes and the expressions of OA-associated genes.

**Results:**

The mRNA levels of COL2A1 and OPN were decreased in knee OA chondrocytes in comparison with those in normal chondrocytes. The OPN deficiency enhanced the senescence and apoptosis of OA chondrocytes and increased the expressions of COL10A1, IL-1*β*, TNF-ɑ, MMP-13, and ADAMTS5 but decreased the expression of COL2A1. Meanwhile, OPN deficiency could result in severe, accelerated OA in vivo, which was also associated with enhanced senescence and apoptosis of chondrocytes and elevated expressions of OA-associated genes.

**Conclusions:**

The findings of this study suggest that the OPN deficiency can result in accelerated OA, which is associated with enhanced senescence and apoptosis of OA chondrocytes and the upregulated expressions of OA-associated genes.

## 1. Introduction

As the most typical form of arthritis, osteoarthritis (OA) is characterized with the degradation of articular cartilage alongside alterations in other joint tissues. With more than 10% of the elderly population affected by this symptomatic disease [1], OA is widely considered to be part of the aging process. The senescence and apoptosis of articular chondrocytes are found to be associated with the pathogenesis of OA [2, 3]. Our earlier studies demonstrated that the elevated SA-*β*-Gal was correlated with the progressive knee OA joint damage [4]. On such basis, the current study was performed for the purpose to further examine the upstream mediator(s) of senescence and apoptosis in cells of OA cartilages.

Osteopontin (OPN), a multifunctional phosphoprotein comprising 300 amino acids, has a critical part in the progression of aging-associated and instability-induced spontaneous OA [5]. An increased expression of OPN and calcium deposits was found enhanced in the osteoarthritic cartilage, while the full-length OPN is mainly posttranslational modified with phosphorylation before it works. The phosphorylation status of OPN can inhibit the formation, growth, and mineralization of hydroxyapatite crystals, while the decrease of OPN and osteocalcin in bones plays an important role in promoting vulnerability to hip fracture [6–8]. OPN is also a pivotal intrinsic factor for regulating cartilage degradation due to its effects on the expression of MMP-13 and proteoglycan loss [9]. Several MMPs have been reported to regulate OPN expression; for example, MMP-9 can cleave an OPN fragment to mediate tumor immune escape, while the MMP-10 can induce the overexpression of OPN in calcific aortic valve stenosis [10, 11]. The elevated expression of OPN, which was found in the joints of OA, is deemed to be correlated with the severity of joint lesions in OA [12–16]. Osteoprotegerin (OPG) takes an important part in maintaining the homeostasis of articular cartilage. Although several studies confirmed that OPN and OPG had a relationship in other diseases, the correlation between OPN and OPG in OA has not been fully elaborated yet [16]. Recently, OPN has been demonstrated to regulate the expressions of ADAMTS4, TIMP-1, TIMP-2, HIF-2*α*, IL-6, and IL-8 [17–20]. Based on a rheumatoid arthritis model of mice, Yumoto et al. [21, 22] suggested that OPN had a significant effect on the destruction of joint cartilage by promoting angiogenesis and inducing chondrocyte apoptosis [21]. In ovariectomized mice, the expressions of both OPN and VEGF increased; in addition, the osteochondral remodeling of the cartilaginous endplate and induced vertebral osteoporosis were found to contribute to angiogenesis and increased porosity on the bone-cartilage surface [23]. Meanwhile, OPN deficiency can suppress the increase in caspase-3 activity induced by TNF-*α* in chondrocytes in the culture. Until recently, the knowledge about the effects of OPN deficiency on the senescence and apoptosis of OA chondrocytes in the pathogenesis of OA is still limited.

Based on the findings above, it is hypothesized that the OPN deficiency is susceptible to OA through the enhancement of chondrocyte senescence and apoptosis and the upregulation of the expressions of OA-associated genes. Accordingly, our study targeted at comprehensively examining the effects of OPN deficiency on the senescence and apoptosis of human OA chondrocytes and on the expressions of OA-associated genes.

## 2. Materials and Methods

### 2.1. Preparation of Human Cartilage and Cell Culture

The present study was carried out in compliance with the recommendations of the Declaration of Helsinki after receiving approval from the ethics committee of XiangYa Hospital. Human OA cartilages were obtained from 20 patients (aged 59–75 years) who underwent total knee joint replacement surgery. OA was diagnosed on the basis of laboratory, clinical, and radiographic examinations (Kellgren–Lawrence grade 4). Normal cartilage tissues were obtained from 5 patients (aged 17–55 years) who undertook the aforementioned knee amputation due to severe trauma. The difference in age between the two groups was not statistically significant.

Primary chondrocytes were extracted from cartilage tissues for culturing. The specimens were minced and incubated with trypsin at 37°C for 15 minutes. Then, the cartilage was processed with Dulbecco's modified Eagle's medium (DMEM; Gibco/Life Technologies, Grand Island, NY, USA) which contained 0.2% collagenase (GIBCO/Invitrogen, Grand Island, NY, USA) at 37°C for 15 hours. The dissociated cells were cultured overnight in DMEM containing 10% fetal bovine serum (DMEM; Gibco/Life Technologies, Grand Island, NY, USA) and 100 U/ml penicillin-streptomycin. After removing nonadherent cells, the adherent cells were incubated in fresh medium on a 6-well plate (1 × 10^4^ cells/well). All procedures were executed with first-passage cells. To characterize the chondrocytes, the type II collagen was confirmed to show a high expression in the OA knee chondrocytes.

The first-passage chondrocytes were then seeded on a 24-well plate at the starting density of 1 × 10^4^ cells/well, until becoming confluent. To characterize the chondrocytes, the type II collagen was confirmed to show a higher expression in normal human knee chondrocytes than in OA knee chondrocytes. In addition, the OPN mRNA levels were analyzed by QPCR for both OA chondrocytes and normal chondrocytes.

Subsequently, the OA chondrocytes were separated into 3 groups: (1) control group: untreated; (2) NC group: treated with control small interfering RNA for 24 hours; and (3) OPN siRNA group: treated with OPN small interfering RNA.

### 2.2. Small Interfering RNA Transfection

OPN and the control of small interfering RNA (siRNA) were obtained from GenePharma (Shanghai, China) and were used to transfect siRNA into human knee OA chondrocytes by following the manufacturer's recommendations. First, the cells were placed in a growth medium without antibiotics on a 6-well plate 1 day before transfection in order to realize 30–50% confluence during the transfection process. Then, complexes of 100 *p*mol of siRNA and Lipofectamine 2000 (Invitrogen, Carlsbad, CA, USA) were prepared and added into each well. After transfection for 24 hours, the complexes were removed and replaced by fresh medium containing 10% FBS. After additional 24 hours, the cells were harvested for RNA and protein extraction.

### 2.3. Western Blot Analysis

The proteins were resolved using SDS-PAGE and transferred to Immobilon polyvinylidene difluoride (PVDF) membranes. Then, the blots were blocked with 4% BSA at room temperature for 1 hour and probed with rabbit anti-human antibodies against OPN, COL2A1, COL10, IL-1*β*, TNF-*ɑ*, MMP-13, and ADAMTS5 (1 : 500) for another 1 hour, at room temperature. Subsequently, the blots were washed for three rounds and incubated with the HRP-conjugated secondary anti-rabbit antibody. The changes in protein expressions were calculated using BioRad Quantity One software, while *β*-actin was applied as an internal control to guarantee identical protein sample loading.

### 2.4. Quantitative Reverse Transcription Polymerase Chain Reaction Analysis (QPCR)

RNA was extracted with TRIzol (Invitrogen), and cDNA synthesis was prepared for 2 *µ*g RNA using SuperScript III from Invitrogen. The relative expressions of mRNA-encoded human COL2A1, COL10, IL-1*β*, TNF-*ɑ*, MMP-13, and ADAMTS5 were examined and analyzed using SYBR Green real-time PCR through an ABI Prism 7700 sequence detection system (Applied Biosystems). The sequences for all the target gene primers and probes were obtained from commercial sources with *β*-actin as internal control (Applied Biosystems). For each individual gene, the mRNA level was quantified by the ΔΔCt method and then normalized to *β*-actin, and the PCR melting curve was examined to evaluate each single, specific product. The difference between the mean Ct of the gene of interest and *β*-actin was denoted by ΔCt, and the difference between ΔCt and Ct of the calibrator sample was denoted by ΔΔCt. The log 2 (−ΔΔCt) value represents the relative level of gene expression.

### 2.5. SA-*β*-Gal Staining and Apoptosis Assay

Positive blue staining of SA-*β*-Gal was chosen as a biomarker for cellular senescence [24]. For detecting SA-*β*-Gal stain, the cells in subconfluent culture were washed with DPSB and fixed for 3–5 minutes in 3.7% formaldehyde first and then washed with DPSB again. Subsequently, the cells were incubated in a CO_2_-free environment overnight with SA-*β*-Gal stain solution (1 mg/ml X-gal, 40 mM citric acid/sodium phosphate, pH 6.0, 5 mM potassium ferrocyanide, 5 mM potassium ferricyanide, 150 mM NaCl, and 2 mM MgCl_2_) at 37°C. Positive staining would appear after 2–4 hours and would be analyzed after 12–14 hours. A total of 500 cells were scored by lightweight microscopy to derive SA-*β*-Gal positive. Apoptotic chondrocytes were quantified through FACS analysis with the cells stained by FITC-conjugated annexin V [25].

### 2.6. In Vivo Rat OA Model

A total of 36 male SD rats (200 ± 20 g, 12 weeks old) were divided into three groups: (1) sham group: the knee of the rat was sham-operated, *n* = 12; (2) control group: the knee joint instability was induced by destabilization of medial meniscus (DMM model) at the left knee and was injected with a nontargeting control plasmid, *n* = 12; and (3) OPN siRNA group: the knee joint instability was induced by DMM at the left knee and was injected with OPN siRNA, *n* = 12 (Cyagen Biosciences Inc., China). Repeat injections were allowed after 3 weeks and 6 weeks, respectively.

The knee joint cartilage of each rat was dissected and analyzed for transfection efficiency and histological evaluation. Standard histologic analysis was conducted using Safranin O as well as hematoxylin and eosin staining, and a series of tests were performed to detect OPN, SA-*β*-Gal, MMP-13, ADAMTS5, collagen type II, and aggrecan. The paraffin sections were routinely deparaffinized after baking for 20 minutes and were hydrated with distilled water and washed for three times (3 minutes each time) with phosphate-buffered saline (PBS). Then, the sections were further processed with complex enzyme digestion for antigen retrieval at room temperature for 15 minutes, incubated with a 3% H_2_O_2_ solution for 10 minutes, and then washed for three times (3 minutes each time) with PBS again. Subsequently, the sections were overlaid at 4°C overnight with antibodies to OPN at 1 : 50 (Proteintech, China), to SA-*β*-Gal at 1 : 50 (Proteintech, China), to MMP-13 at 1 : 50 (Proteintech, China), to ADAMTS5 at 1 : 50 (Abcam, China), to collagen II at 1 : 50 (Proteintech, China), and to aggrecan at 1 : 100 (Proteintech, China). The prepared samples were then incubated with 30 *µ*L of biotinylated goat anti-rabbit secondary antibody for 30 minutes and washed for three times (5 minutes each time) with PBS and were further stained using 50 *µ*L of the 3, 3′-diaminobenzidine solution with appropriate termination and rinsed with tap water for 3 hours. Thereafter, counterstaining was performed using hematoxylin for 5 minutes. Then, each section was dehydrated by a graded series of alcohol for 10 seconds and was cleared using xylene and mounted with neutral balsam. The Olympus photomicroscope was engaged to photograph the sections. The relative OPN distribution of cartilage tissues could be visualized and quantified as optical density (OD). A semiquantitative assessment was performed on the mean optical density of OPN expression on the basis of scanned autoradiograms with ImageJ. Grayscale images were acquired and converted to absorbance units, in order to analyze the area from cartilage surface to the cartilage-bone junction. Analysis of chondrocyte apoptosis in cartilage tissues was conducted by the TUNEL assay using a kit manufactured by Roche Diagnostics (Nanjing KeyGen Biotech., China).

### 2.7. Statistical Analysis

All the values were presented as mean ± SD. SPSS for MS Windows (version 16.0) was utilized for statistical analysis. One-way analysis of variance (ANOVA) was applied to compare the means among different groups. Pearson's correlation and linear regression were used to examine the correlation between OARSI scores and the optical density of proteins in the articular cartilage. *p* value ≤0.05 was deemed statistically significant.

## 3. Results

### 3.1. The Expression Levels of OPN and COL2A1 Decreased in Human OA Chondrocytes

**Figure 1:**
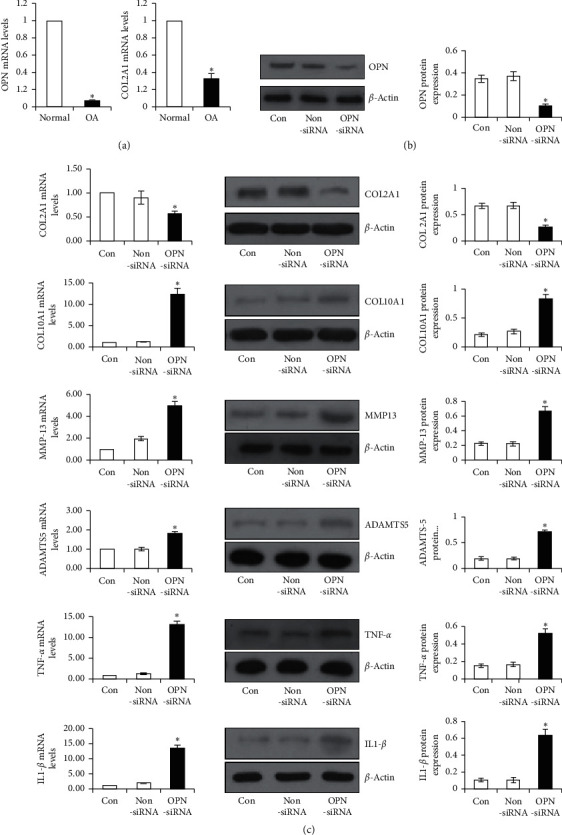
Expressions of OPN and COL2A1 of the human OA chondrocytes and effects of OPN siRNA on the expression of osteoarthritis-associated genes. (a) The mRNA levels of OPN and COL2A1 in OA chondrocytes and in normal chondrocytes (^*∗*^*p* < 0.05 vs normal). (b) Western blot analysis was developed to evaluate the knockdown efficiency of OPN siRNA transfection. (c) Effects of OPN siRNA on the gene levels and protein levels of COL2A1, COL10A1, IL-1*β*, TNF-*ɑ*, MMP-13, and ADAMTS5 (^*∗*^*p* < 0.05 vs control or non-siRNA).

The mRNA levels of OPN and COL2A1 were compared between human normal chondrocytes and OA chondrocytes in order to evaluate the expressions of OPN and cartilage matrix-related genes in chondrocytes. The real-time PCR indicated that the mRNA levels of OPN and COL2A1 decreased significantly in OA chondrocytes relative to those in normal chondrocytes (Figure 1(a)).

### 3.2. The Effects of OPN siRNA on the Expression of Osteoarthritis-Associated Genes and the Senescence and Apoptosis of OA Chondrocytes

Western blot analysis was conducted to evaluate the knockdown efficiency of OPN siRNA transfection. The results suggested that the expressions of OPN in human OA chondrocytes were inhibited by the OPN-specific siRNA transfection (Figure 1(b)).

**Figure 2:**
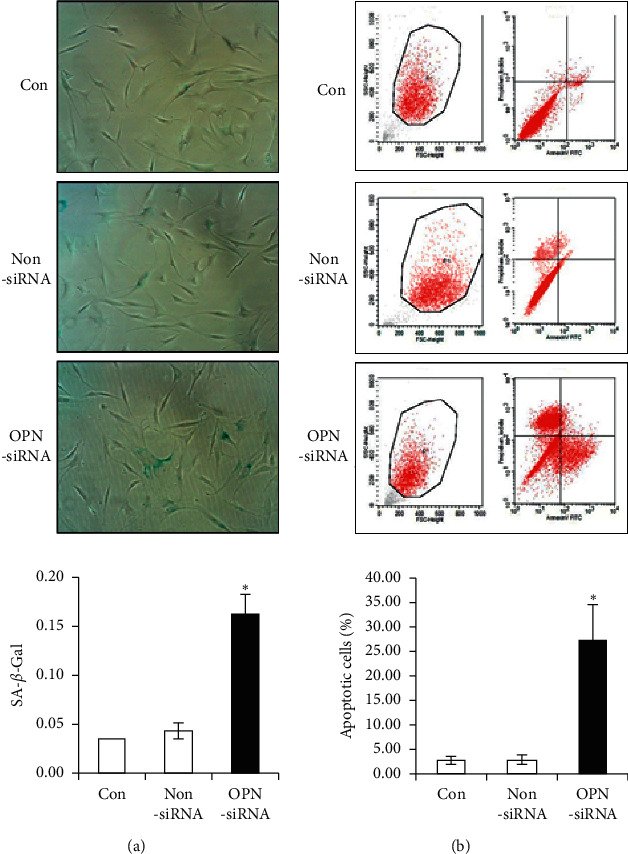
Effects of OPN siRNA on the senescence (a) and apoptosis (b) of OA chondrocytes (^*∗*^*p* < 0.05 vs control or non-siRNA).

To examine the function of OPN on OA chondrocytes, the mRNA levels and protein expressions of COL2A1, COL10A1, IL-1*β*, TNF-*ɑ*, MMP-13, and ADAMTS5 were compared between OPN-knockdown OA chondrocytes and controls. The downregulation of OPN resulted in a reduction in the COL2A1 expression (both at the gene and protein levels) and an increase in the expressions of COL10A1, IL-1*β*, TNF-*ɑ*, MMP-13, and ADAMTS5 (both at the gene and protein levels) in comparison with the control group and NS group (Figure 1(c)). Meanwhile, the SA-beta-Gal expression (Figure 2(a)) and apoptotic cell percentage (Figure 2(b)) were increased in the OA chondrocytes treated with OPN siRNA in comparison with the control and NS groups.

### 3.3. OPN Deficiency Results in Severe, Accelerated Osteoarthritis *In Vivo*

**Figure 3:**
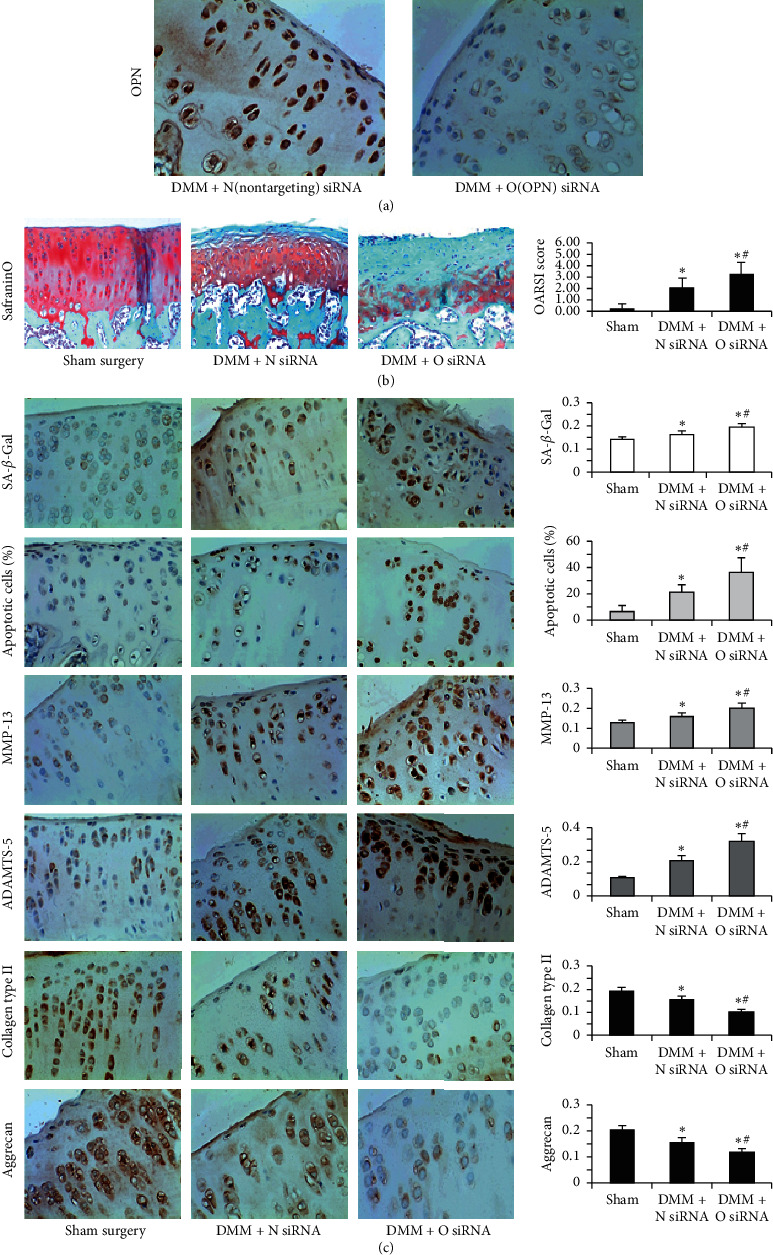
In vivo study of the role of OPN deficiency in OA. (a) Immunohistochemistry was employed to evaluate the knockdown efficiency of OPN siRNA. (b) Safranin O staining of cartilage was performed and OARSI scores were compared among the three groups. (c) The expressions of SA-*β*-Gal, MMP-13, ADAMTS5, collagen type II, and aggrecan and the chondrocyte apoptosis percentage were compared among the three groups (^*∗*^*p* < 0.05 vs sham, ^#^*p* < 0.05 vs DMM + nontargeting siRNA).

To examine the effect of OPN in vivo, a loss of OPN function strategy was developed by injecting OPN siRNA into the knee joints of rats in a DMM model. The prepared rat samples were then compared with samples treated by nontargeting siRNA injection. As expected, the loss of OPN expression in articular chondrocytes in the DMM group with OPN siRNA injection was confirmed by immunohistochemistry (Figure 3(a)). Safranin O and fast green staining indicated that the sham surgery did not show matrix depression (Figure 3(b)). The DMM rats with nontargeting siRNA injection demonstrated retention of the articular surface layer, but loss of Safranin O staining was observed at the same time (Figure 3(b)). The DMM rats with OPN siRNA injection showed a larger loss of proteoglycans, as well as loss of cellularity and destruction in certain regions of the articular cartilage (Figure 3(b)). Based on the OARSI cartilage OA histopathology scoring system, the OARSI score was remarkably increased in the DMM group relative to that in the sham surgery control group. Furthermore, the OARSI score was significantly higher in the DMM group with OPN siRNA injection than in the DMM group with nontargeting siRNA injection (Figure 3(b)).

### 3.4. OPN Deficiency Results in Accelerated OA Associated with Aberrant Chondrocyte Senescence and Apoptosis

Compared with the sham-operated group, the DMM group showed a higher expression of SA-*β*-Gal and percentage of apoptotic cells. Meanwhile, higher expression of SA-*β*-Gal and percentage of apoptotic cells were also observed in the DMM group with OPN siRNA injection in comparison with the DMM group with nontargeting siRNA injection (Figure 3(c)). Interestingly, the expression of SA-*β*-Gal and percentage of apoptotic cells showed a positive correlation with the OARSI score in the articular cartilage (*r* = 0.72, *p* < 0.001 and *r* = 0.81, *p* < 0.001, respectively).

### 3.5. OPN Deficiency Results in Accelerated OA Associated with the Upregulation of the Expression of Osteoarthritis-Associated Genes

In view of that, matrix metalloproteinase (MMP)-13 is one of the important catabolic factors for OA; in this study, the expression of MMP-13 was determined by immunohistochemistry. The results revealed an increased MMP-13 expression in the cartilage in the OPN siRNA injection group in comparison with that in the nontargeting siRNA injection group (Figure 3(c)). Furthermore, the expression of ADAMTS5 (another key catabolic factor for OA) was increased while the expressions of collagen type II and aggrecan (two major anabolic components of the ECM in the articular cartilage) were decreased in the OA cartilage in the OPN siRNA injection group in comparison with that in the nontargeting siRNA injection group (Figure 3(c)). These results suggested that the rats with OPN siRNA injection subjected to OA surgery exhibited enhanced catabolic activity and accelerated chondrocyte loss in the articular cartilage. Interestingly, the expressions of MMP-13 and ADAMTS5 showed a positive correlation with the OARSI score in the articular cartilage (*r* = 0.78, *p* < 0.001 and *r* = 0.83, *p* < 0.001, respectively).

## 4. Discussion

Recent studies have reported the existence of a definite correlation between the degree of cartilage damage and the senescence and apoptosis of chondrocytes [26–28]. Wang et al. [29] claimed that the changes in gene expression in OPN signaling pathways might explain the enhanced cell adhesion, survival, proliferation, and migration, as well as the augmented inflammation response and the attenuated apoptosis in the process of liver regeneration. Tahir et al. [30] revealed that the A CD153 + T follicular cell population featured with cell senescence played a key role in lupus pathogenesis through OPN production. These findings suggest that OPN may account for the change of cell-apoptosis and cell-senescence features. Therefore, to determine the function of OPN in OA chondrocytes, our study mainly focused on the senescence and apoptosis of OA chondrocytes and the expressions of OA-associated genes. It has been reported that OPN has an important effect on the destruction of joint cartilage by promoting angiogenesis and inducing chondrocyte apoptosis in the mice of the rheumatoid arthritis model [21]. However, the knowledge about the effects of OPN deficiency on the senescence and apoptosis of OA chondrocytes in the pathogenesis of OA is still very limited. The results of this study, in the first place, demonstrated that OPN deficiency could accelerate the senescence and apoptosis of OA chondrocytes.

OA is a progressive degenerative joint disease triggered by tearing and wearing on the articular surface. The osteoarthritic changes in the articular cartilage involve the progressive proteolytic degradation of the related extracellular matrix (mainly composed of COL2A1 and aggrecan). In this study, the downregulation effect of OPN deficiency on the expression level of COL2A1 implied that the OPN deficiency in OA chondrocytes could decrease extracellular matrix deposition. In addition to the stimulatory effects on cartilage matrix formation, the upregulation effect of OPN deficiency on the expression level of COL10A1 suggested further induction of the phenotype of OA chondrocytes, as the hypertrophic differentiation characterized by the increase of COL10A1 expression levels is a hallmark of OA. In view of the complexity of OA etiology, both biological and biomechanical factors (e.g., mechanical strain and inflammatory cytokines) can affect the homeostatic balance between anabolic and catabolic factors in the joint, which may eventually lead to the damage of articular cartilage. Specifically, the synovial cells and articular chondrocytes can generate a variety of catabolic factors, including aggrecanases (e.g., ADAMTS4 and -5), matrix metalloproteinases (e.g., MMP-1 and -13), and proinflammatory factors/cytokines (e.g., IL-1, IL-6, and TNF-*α*), which all contribute to joint destruction in OA [31, 32]. By investigating the role of OPN as a regulator of matrix degradation in the cartilage, the present study demonstrated that the OPN deficiency-induced alterations of the proinflammatory cytokines TNF-*α* and IL-1*β*, which were reported to be upregulated in OA [31, 32]. These proinflammatory cytokines can enhance the inflammatory process and induce several matrix-degrading enzymes and are thus regarded as key regulators of cartilage degradation. Moreover, it was also found that OPN deficiency induced the expressions of MMP-13 and ADAMTS5 in OA chondrocytes, suggesting that OPN mediated and modulated ECM degradation.

This study established a mouse OA model, which was used to examine the role of OPN deficiency. A recent study claimed that the OPN deficiency exacerbated both aging-related and instability-induced OA, and OPN acted as a pivotal intrinsic regulator of cartilage degradation through its effects on MMP-13 expression and proteoglycan loss [9]. Comparatively, the present study indicated that the OPN deficiency resulted in severe, accelerated OA in vivo and induced elevated expressions of SA-*β*-Gal and percentage of apoptotic cells in OA chondrocytes. It is noteworthy that the expression of SA-*β*-Gal and the percentage of apoptotic cells in the OA articular cartilage showed a positive correlation with the OARSI score. In addition, OPN deficiency upregulated the expressions of OA-associated genes and resulted in accelerated OA associated with the upregulation of the expression of OA-associated genes. These findings provided support to our in vitro experiments.

The present study also showed that the mRNA levels of OPN in OA chondrocytes were decreased significantly relative to that in normal chondrocytes. However, several published studies and our earlier studies suggested that the increased OPN expression in the synovial fluid, cartilage, and synovium of OA patients was correlated with the severity of joint lesion [12–16]. Furthermore, our previous results implied that OPN could regulate the expressions of ADAMTS4, HIF-2*α*, TIMP-1, TIMP-2, IL-6, and IL-8 and might have a protective effect on human OA chondrocytes against aggrecan degradation through the suppression of ADAMTS4 expression [17–20]. Similarly, this study also revealed that OPN deficiency worsened the severity of OA associated with enhanced senescence and apoptosis of OA chondrocytes and increased the expressions of OA-associated genes. Thus, elevated OPN expression can be considered as an adaptive response to protect cells from the catabolic and inflammatory environment in the development of OA. Any failure of this response can probably lead to further progression of the degenerative process. These findings as well as our results suggested that OPN might be responsible for repairing the human osteoarthritic cartilage, and the remodeling of cartilage played a certain role in elevating the OPN level. Many studies had confirmed that OPN could affect COL2A1, COL10A1, IL-1*β*, TNF-*α*, MMP-13, and ADAMTS5 gene expressions, and we also had tested the above gene expressions. Several genes were the key roles in OA, but they were not the direct target of OPN. OPN should bind to its receptor including integrin and CD44 at first and subsequently, activate some pathways such as NF- *κ* B pathway to regulate the OA-associated gene expressions [33].

In conclusion, OPN deficiency in OA chondrocytes increases the expressions of OA-related genes, accelerates apoptosis and cellular senescence, and enhances hypertrophic-like changes. Our findings suggest that OPN plays a crucial role in protecting human chondrocytes from the inflammatory environment. In view of this, our study provides important references for elaborating the pathophysiology of OA and points out a potential target for its treatment.

## Data Availability

The data used to support the findings of this study are available from the corresponding author upon request.

## References

[1] Cross M., Smith E., Hoy D. (2014). The global burden of hip and knee osteoarthritis: estimates from the global burden of disease 2010 study. *Annals of The Rheumatic Diseases*.

[2] Rahmati M., Nalesso G., Mobasheri A. (2017). Aging and osteoarthritis: central role of the extracellular matrix. *Ageing Research Reviews*.

[3] Yang H., Wen Y., Zhang M. (2020). MTORC1 coordinates the autophagy and apoptosis signaling in articular chondrocytes in osteoarthritic temporomandibular joint. *Autophagy*.

[4] Gao S. G., Zeng C., Li L. J. (2016). Correlation between senescence-associated beta-galactosidase expression in articular cartilage and disease severity of patients with knee osteoarthritis. *International Journal of Rheumatic Diseases*.

[5] Matsui Y., Iwasaki N., Kon S. (2009). Accelerated development of aging-associated and instability-induced osteoarthritis in osteopontin-deficient mice. *Arthritis & Rheumatology*.

[6] Halling L. C., Ek-Rylander B., Krumpel M. (2017). Bone alkaline phosphatase and tartrate-resistant acid phosphatase: potential Co-regulators of bone mineralization. *Calcified Tissue International*.

[7] Martinez-Calleja A., Velasquillo C., Vega-Lopez M. (2014). Osteopontin expression and localization of Ca++ deposits in early stages of osteoarthritis in a rat model. *Histology And Histopathology*.

[8] Tanaka S., Narusawa K., Onishi H. (2011). Lower osteocalcin and osteopontin contents of the emoral head in hip fracture patients than osteoarthritis patients. *Osteoporosis International*.

[9] Matsui Y., Iwasaki N., Kon S. (2009). Accelerated development of aging-associated and instability-induced osteoarthritis in osteopontin-deficient mice. *Arthritis & Rheumatology*.

[10] Matilla L., Roncal C., Ibarrola J. (2020). A role for MMP-10 (matrix metalloproteinase-10) in calcific aortic valve stenosis. *Arteriosclerosis, Thrombosis, and Vascular Biology*.

[11] Shao L., Zhang B., Wang L. (2017). MMP-9-cleaved osteopontin isoform mediates tumor immune escape by inducing expansion of myeloid-derived suppressor cells. *Biochemical and Biophysical Research Communications*.

[12] Pullig O., Weseloh G., Gauer S. (2000). Osteopontin is expressed by adult human osteoarthritic chondrocytes: protein and mRNA analysis of normal and osteoarthritic cartilage. *Matrix Biology*.

[13] Honsawek S., Tanavalee A., Sakdinakiattikoon M. (2009). Correlation of plasma and synovial fluid osteopontin with disease severity in knee osteoarthritis. *Clinical Biochemistry*.

[14] Gao S. G., Li K. H., Zeng K. B. (2010). Elevated osteopontin level of synovial fluid and articular cartilage is associated with disease severity in knee osteoarthritis patients. *Osteoarthritis Cartilage*.

[15] Xu M., Zhang L., Zhao L. (2013). Phosphorylation of osteopontin in osteoarthritis degenerative cartilage and its effect on matrix metalloprotease 13. *Rheumatology International*.

[16] Liu Y., Ge J., Chen D. (2016). Osteoprotegerin deficiency leads to deformation of the articular cartilage in femoral head. *Journal of Molecular Histology*.

[17] Gao S. G., Zeng C., Song Y. (2015). Effect of osteopontin on the mRNA expression of ADAMTS4 and ADAMTS5 in chondrocytes from patients with knee osteoarthritis. *Experimental and Therapeutic Medicine*.

[18] Cheng C., Zhang F. J., Tian J. (2015). Osteopontin inhibits HIF-2*α* mRNA expression in osteoarthritic chondrocytes. *Experimental and Therapeutic Medicine*.

[19] Zhang F. J., Yu W. B., Luo W. (2014). Effect of osteopontin on TIMP-1 and TIMP-2 mRNA in chondrocytes of human knee osteoarthritis in vitro. *Experimental and Therapeutic Medicine*.

[20] Yang Y., Gao S. G., Zhang F. J. (2014). Effects of osteopontin on the expression of IL-6 and IL-8 inflammatory factors in human knee osteoarthritis chondrocytes. *European Review for Medical and Pharmacological Sciences*.

[21] Yumoto K., Ishijima M., Rittling S. R. (2002). Osteopontin deficiency protects joints against destruction in anti-type II collagen antibody-induced arthritis in mice. *Proceedings of the National Academy of Sciences of the United States of America*.

[22] Yumoto K., Nifuji A., Rittling S. R. (2012). Osteopontin deficiency suppresses tumor necrosis factor-*α*-induced apoptosis in chondrocytes. *Cartilage*.

[23] Xiao Z. F., He J. B., Su G. Y. (2018). Osteoporosis of the vertebra and osteochondral remodeling of the endplate causes intervertebral disc degeneration in ovariectomized mice. *Arthritis Research & Therapy*.

[24] Dimri G. P., Lee X., Basile G. (1995). A biomarker that identifies senescent human cells in culture and in aging skin in vivo. *Proceedings of the National Academy of Sciences of the United States of America*.

[25] Kim S. J., Ju J. W., Oh C. D. (2002). ERK-1/2 and P38 kinase oppositely regulate nitric oxide-induced apoptosis of chondrocytes in association with P53, caspase-3, and differentiation status. *Journal of Biological Chemistry*.

[26] Hwang H. S., Kim H. A. (2015). Chondrocyte apoptosis in the pathogenesis of osteoarthritis. *International Journal of Molecular Sciences*.

[27] Takayama K., Kawakami Y., Lee S. (2014). Involvement of ERCC1 in the pathogenesis of osteoarthritis through the modulation of apoptosis and cellular senescence. *Journal of Orthopaedic Research*.

[28] Kim K. M., Kim J. M., Yoo Y. H. (2012). Cilostazol induces cellular senescence and confers resistance to etoposide-induced apoptosis in articular chondrocytes. *International Journal of Molecular Medicine*.

[29] Wang G., Chen S., Zhao C. (2016). Gene expression profiles predict the possible regulatory role of OPN-mediated signaling pathways in rat liver regeneration. *Gene*.

[30] Tahir S., Fukushima Y., Sakamoto K. (2015). A CD153+CD4+ T follicular cell population with cell-senescence features plays a crucial role in lupus pathogenesis via osteopontin production. *Journal of Immunology*.

[31] Benito M. J., Veale D. J., FitzGerald O. (2005). Synovial tissue inflammation in early and late osteoarthritis. *Annals of the Rheumatic Diseases*.

[32] Pelletier J. P., Martel-Pelletier J., Abramson S. B. (2001). Osteoarthritis, an inflammatory disease: potential implication for the selection of new therapeutic targets. *Arthritis & Rheumatology*.

[33] Li Y., Jiang W., Wang H. (2016). Osteopontin promotes expression of matrix metalloproteinase 13 through NF-kappaB signaling in osteoarthritis. *Biomed Research International*.

